# A comparative study on green synthesis and characterization of Mn doped ZnO nanocomposite for antibacterial and photocatalytic applications

**DOI:** 10.1038/s41598-024-58393-0

**Published:** 2024-03-29

**Authors:** Murtaza Hasan, Qiang Liu, Ayesha Kanwal, Tuba Tariq, Ghazala Mustafa, Sana Batool, Mansour Ghorbanpour

**Affiliations:** 1 Faculty of Medicine, Dalian University of Technology, Dalian, 116024 People’s Republic of China; 2 Department of Biotechnology, Faculty of Chemical and Biological Sciences, The Islamia University of Bahawalpur, Bahawalpur, 63100 Pakistan; 3 Depatment of Plant Sciences, Faculty of Biological Sciences, Quaid-i-Azam University, Islamabad, 45320 Pakistan; 4 Key Laboratory of Horticultural Plant Growth and Development, Ministry of Agriculture, Department of Horticulture, Zhejiang University, Hangzhou, 310058 China; 5 Department of Medicinal Plants, Faculty of Agriculture and Natural Resources, Arak University, Arak, 38156-8-8349 Iran; 6 Institute of Nanoscience and Nanotechnology, Arak University, Arak, 38156-8-8349 Iran

**Keywords:** Biosynthesis, Mn-doped ZnO nanoparticles, Photocatalytic, Bio-reducing agent, Methylene blue dye, Antimicrobial activity, Physiology, Plant sciences, Chemistry

## Abstract

Biological and green synthesis of nanomaterial is a superior choice over chemical and physical methods due to nanoscale attributes implanted in a green chemistry matrix, have sparked a lot of interest for their potential uses in a variety of sectors. This research investigates the growing relevance of nanocomposites manufactured using ecologically friendly, green technologies. The transition to green synthesis correlates with the worldwide drive for environmentally sound procedures, limiting the use of traditional harsh synthetic techniques. Herein, manganese was decorated on ZnO NPs via reducing agent of *Withania-*extract and confirmed by UV-spectrophotometry with highest peak at 1:2 ratio precursors, and having lower bandgap energy (3.3 eV). XRD showed the sharp peaks and confirms the formation of nanoparticles, having particle size in range of 11–14 nm. SEM confirmed amorphous tetragonal structure while EDX spectroscopy showed the presence of Zn and Mn in all composition. Green synthesized Mn-decorated ZnO-NPs screened against bacterial strains and exhibited excellent antimicrobial activities against gram-negative and gram-positive bacteria. To check further, applicability of synthesized Mn-decorated Zn nanocomposites, their photocatalytic activity against toxic water pollutants (methylene blue (MB) dye) were also investigated and results showed that 53.8% degradation of MB was done successfully. Furthermore, the installation of green chemistry in synthesizing nanocomposites by using plant extract matrix optimizes antibacterial characteristics, antioxidant and biodegradability, helping to build sustainable green Mn decorated ZnO nanomaterial. This work, explains how biologically friendly Mn-doped ZnO nanocomposites can help reduce the environmental impact of traditional packaging materials. Based on these findings, it was determined that nanocomposites derived from biological resources should be produced on a wide scale to eradicate environmental and water contaminants through degradation.

## Introduction

Infectious illnesses, which can be transmitted from one person to another, can have a significant impact on large groups of individuals and may result in an epidemic^1^. To prevent the initial stages of infection, early diagnosis is crucial^2^, which can be achieved through in-vitro diagnostics. Treating infections caused by pathogenic bacteria can be challenging, as there are often limited or no effective antimicrobials available. The emergence and spread of multidrug-resistant (MDR) strains of pathogenic bacteria pose a significant threat to public health^3^. Infectious diseases are a global danger that has a financial impact, and every year, they cause the deaths of approximately 17 million people^4^. Infections are more prevalent in underdeveloped and emerging countries, with malnourished and immune-compromised populations being particularly vulnerable^5,6^.

Nanotechnology has added value to every aspect of human life and has led to the development of many new advanced items for the betterment of human life^7–10^. The study of synthesizing and using materials with unique features at nanoscale dimensions, ranging from 1 to 100 nm^11^, has opened up new applications^12,13^. As technology has advanced, various physical and chemical methods have been used to create nanoparticles such as silver^14^, copper^15^, gold, zinc oxides^16^, iron oxides, calcium oxides, titanium oxide, manganese oxides^17^, carbon nanotubes (CNTs)^18^, mesoporous silica nanoparticles, and quantum dots^12^. Due to their unique physical features, they have a wide range of uses in biological sciences^19^, including biotechnology^20^, electronics, food, medicine, and catalysis^21,22^.

Chemical and physical methods for synthesizing nanoparticles are poisonous, expensive, and dangerous to the environment they are costly, toxic, and require higher energy to complete the process^23,24^. On the other hand, plants offer a superior choice for producing nanoparticles through the green synthesis effect^25^, which produces non-toxic particles^26,27^. Green nanoparticle synthesis has a high yield, cheap cost, short reaction time, and eco-friendly process, and nonetheless^28,29^. One of the most common plants employed in the production of nanoparticles is *W. coagulans*. It is an old plant that belongs to the Solanaceae family^30^. It is most widespread in the Mediterranean's east, although it can also be found in South Asia. *W. coagulans* consists of rich organic compounds like withanoside, withanolide, and withaferin can act as a strong reducing agent for the synthesis of the nanocomposite. The novelty of this plant extract *W. coagulans* is an effective therapeutic nature as well as a better photocatalytic agent as reported by^31,32^. This plant extract, derived from *W. coagulans* (paneer) fruits, plays a crucial role in reducing metal precursors and facilitating the formation of nanoparticles. The use of *W. coagulans* in this context is innovative and environmentally friendly, offering a sustainable approach to nanomaterial synthesis^32,33^. This green synthesis method stands out as a safer and more eco-conscious alternative to traditional chemical and physical methods, aligning with the principles of green chemistry. The specific concentration of the plant extract, along with its confirmation of composites, adds to the novelty, showcasing its potential for eco-friendly nanomaterial production^34^.

Amongst, different bio-elements manganese (Mn) is a vital component of the human body and however, it is required in trace amounts. It works as adhesion and propagation and regulates bone metabolism and cellular proliferation^35,36^. Because of their unique physical and chemical properties, Mn oxides can be used in molecular sieves, photovoltaic cells, light emitting diodes, drug delivery ion sieves, and a variety of other applications, including imaging contrast agents, magnetic hard disks, food sciences^37,38^ and water purification. It also increases the flexibility of the Zn alloys which ultimately affects human development, maturation, and immune function because it is required for hundreds of enzymatic reactions^39–41^.

Zinc and manganese as nanocomposites with other metals have been claimed to play a role in biomedical applications. They have the highest stability against Bacterial pathogens and efficiently kill the bacterial colonies^42^. The mechanism of action is the attachment of nanomaterial to the cell membrane causing a series of fluctuations in the internal environment. It is caused by disturbing the ion gradient, stopping protein synthesis, mitochondrial dysfunction, and causing apoptosis^43–45^.

Photophysical characteristics of luminous Zn campsites are of greater quality with novel surface modifications and quantum yield. There are many reports on the preparation of manganese (Mn) based zinc nanocomposites by using sole gel process, microwave, radiation etc. but there is a dire need for scientific research on the eco-friendly synthesis of Mn-doped ZnO nanoparticles and their application in the biological arena. So, keeping in view all of the data mentioned above, this study was planned for green synthesis of the composite, characterization and its application in the biological field.

## Materials and methods

### Materials

Distilled water was utilized to prepare the solution. Analytical grade chemicals, including Zinc (II) acetate dihydrate ACS reagent (≥ 98%), Manganese (II) chloride tetrahydrate ACS reagent (≥ 99%), Sodium hydroxide ACS reagent (≥ 97%), and Methylene blue Sigma-Aldrich (≥ 95%), are used in tests to maintain pH

### Preparation of bio reducing agent

*W. coagulans* obtained from local market of Bahawalpur, Pakistan. The fruits of *W. coagulans* (paneer) were washed with tap water two times and then with distilled water. Afterward, plant fruits were dried under the sunlight and then crushed into fine powder Fig. S1.

### Mn-doped ZnO nanocomposites preparation

For the preparation of Mn-doped ZnO nanoparticles, different concentrations of Zn and Mn were used such as 1:1, 1:2, 4:1, and 2:1 using *W. coagulans* extract, and the aqueous salt solution was prepared by dissolving 0.2 g zinc (II) acetate dihydrate [Zn(CH_3_CO_2_)_2_.2H_2_O] and 0.2 g manganese(ӀӀ) chloride tetrahydrate (MnCl_2_.4H_2_O) powder in 50 mL distilled water with stirring of 10–15 min on a hotplate was carried out. Then 20 mL extract *W. coagulans* filtered extract was added at room temperature by maintaining the pH at 12 with 2 M sodium hydroxide (NaOH) solution; later on, continue stirring, for 5 h until the color changes from light brown to dark brown. Finally, the solution was sonicated for 20–30 min at room temperature followed by the centrifugation at 6000 rpm at 15 °C for 10 min and placed in the incubator for drying at 80 °C overnight. The powder of Mn-doped ZnO nanoparticles was synthesized as the final product Fig. S2. The same protocol was followed to prepare the composites with different molar ratios of 1:2, 4:1, and 2:1 by varying pH values 8 and 10.

To prepare Mn-doped ZnO nanoparticles, add 0.2 molar Zinc acetate salt in 50 mL distilled water and stir it for 10 to 15 min on the hot plate with continuous heating at 70–80 °C. After that, manganese chloride salt was added to the zinc acetate solution and stirred further for 10 to 15 min on continuous heating. Next, add 20 mL plant extract to this solution and maintain the pH at 12 with sodium hydroxide (NaOH). As soon as pH is maintained at 12, the solution color is turned dark brown and stirred for 5 h with continuous heating at a fixed temperature. It was noted after the addition of the extract mixture to the reaction mixture and heated for 5 h, it changed from dark brown to light brown (Fig. S2a–d). The change in color shows evidence of the reduction process, which shows the synthesis of Mn-doped ZnO nanoparticles. The results show different colors in Fig. S2a–d and the synthesis of these nanoparticles is given in Fig. S3.

### Characterization studies of Mn-doped ZnO nanocomposites

#### UV- visible spectrometry

Optical spectra of Mn-doped ZnO Nanocomposites have been recorded to confirm the synthesis of the nanomaterial. UV–Vis absorption spectra were recorded at room temperature using a UV spectrophotometer (Epoch-BioTek Instruments USA) operated at a resolution of 1 nm in the wavelength ranges from 300 to 800 nm. The spectra were obtained after every 30-min difference in all aliquots as a function of time retortion for the optimization process.

#### X-ray diffraction analysis (XRD)

The size and structural features of Mn-doped ZnO composites were measured using an X-ray diffraction diffractometer employing CuKα having λ = 1.5406 Å (Bruker-D8) at 40 kV, 30 mA current, 0.3 s per count, and a scanning range of 20–80°.

#### Scanning electron microscopy (SEM)

Surface morphology was examined utilizing a HITACHI S4800 emission scanning electron microscope (SEM) with HORIBA EMAX energy dispersive X-ray (EDAX) for elemental composition analysis.

#### Fourier transform infrared spectroscopy (FTIR)

To identify functional subsets of Mn-doped ZnO and how they interact between nanoparticles and the *W. coagulant* capping agent. Biologically produced samples from *W. coagulant* were air-dried before analysis. The powder sample was subjected and characterized by using a Thermos Scientific spectrophotometer with a resolution of 4 cm^−1^ in the 600–4000 cm^−1^ range.

### Photocatalytic degradation

The photocatalytic activity of Mn-doped ZnO nanocomposites was prepared by the green method with different molar ratios and pH and further evaluated for degradation of methylene blue (MB) dye under sunlight. At the initial stage of the experiment, 20 mg of the synthesized Mn-doped ZnO nanocomposite of each sample was dissolved in the 50 mL dye solution having a concentration of 5 mg/L at pH 8. To achieve the adsorption equilibrium, the solution was vigorously stirred in the dark for about 30 min before being exposed to sunlight. The reaction continued; and 5 mL of solution was removed at 30 min intervals. The resulting solution was centrifuged on a UV–vis spectrometer to separate the catalyst and trace absorption spectra. MB dye solution has a maximum absorption wavelength of 660 nm and the following equation measures the percentage of catalyst degradation:

$${\text{Degradation}}\,{\text{efficiency}}\,\left( {\text{D}} \right)\, = \, \left[ {\left( {{{C_o}}{-}{{ C_t}}} \right) \, /{\text{ Co}}} \right]\, \times \,100\% ,$$

where C_o_ = initial concentration of dye, C_t_ = final concentration of dye.

### Antimicrobial activity

The disc diffusion method was used to analyze the antibacterial efficiency of biologically prepared Mn-doped ZnO nanocomposites against *E. coli* and *S. aureus*. To do this activity, combine 100 mL distilled water with 1.3 g nutrient broth, autoclave the broth solution, pour 20 mL into a conical flask, add 100 mL bacteria, and shake overnight at 37 °C. When the solution becomes cloudy, which indicates that bacterial growth has been completed. After that, dissolve 3.6 g nutrient agar in the 300 mL distilled water, autoclave it, pour it into the petri plates, and wait until it solidifies. After petrification, 50 μL of the bacterial solution was added to plates, dispersed evenly around the plate with a spreader, and left for 45 min to allow bacteria to settle on the agar plates. Filter paper discs were plunged and added in different proportions of Mn-doped ZnO nanocomposites (10, 20, and 40%).

After the bacteria on the plates have stabilized, take the dipped discs and place them on the plates. After completing these steps, laminate the Petri plates with parafilm to avoid dehydration and contamination, and incubate for 24 h at 37 °C. Check bacterial growth and measure the zone of inhibitions demonstrating antibacterial activity of biologically Mn-doped ZnO nanoparticles.

### Statistical analysis

All the data was analyzed in triplicate and statistically analysis was performed.

### Statement on experimental research and field studies on plants

The cultivated plants sampled comply with relevant institutional, national, and international guidelines and domestic legislation of Pakistan.

## Results and discussions

The main goal of this research is to optimized of bioreducing agent, precursors of Mn-doped ZnO, temperature and their biological applications.

**Figure 1:**
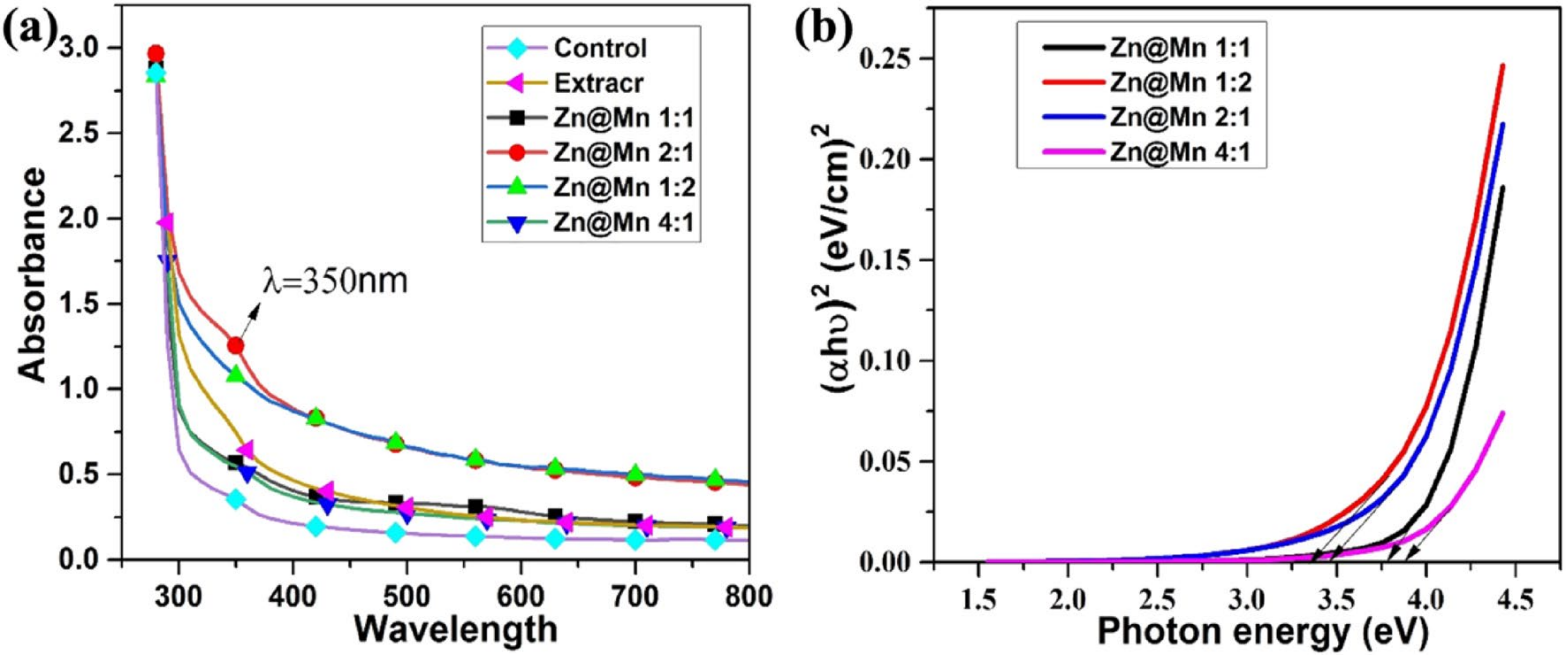
The comparative UV spectra of biologically synthesized Mn-doped ZnO nanoparticles with optimization (**a**) Band gap of Mn-doped ZnO Nanocomposites (**b**).

Utilizing UV–vis spectra of control and sample solutions with wavelengths between 200 and 800 nm, it was possible to prove the existence of stable nanocomposites. The biologically fabricated sample having zinc manganese ratio 2:1 with a pH of 12 has shown an excellent absorption band at around 350 nm in the UV region as shown in Fig. 1a. In contrast, the rest of the whole ratio for preparation of composites having different ratios lacks a peak as observed in the control sample which has no peak because due to absence of a reducing agent Fig. 1a. This result showed that plant extract with specific concentration works and confirmed the composites as previously reported^11,46, 47^.

The optical band gap (Eg) value is determined by measuring the total absorption, which is associated with the excitation of an electron from the valance band to the conduction band. Davis and Mott said that in the case of the high absorption area of a semiconductor, the relationship between the incident photon energy (hυ) and the absorption coefficient (α) is determined using Tauc's law. The optical energy band gap shows the energy needed to excite an electron from its lower energy band to the higher energy band. The optical energy band gap is considered a crucial variable when studying optics for optoelectronics-related technological applications. Ingrown samples, the optical band gap (Eg) was calculated using Tauc equations is given as folls: $ah\upsilon =A{(h\upsilon -{E}_{g})}^{n}$.

Here the symbol "A" stands for constant (energy independent), "υ" (Hz) stands for the frequency of light, "h" stands for Planck's constant (6.626 $\times$ 10^–34^ m^2^ Kg/s, "*E*g" stands for the optical energy band gap, "α" stands for the absorbance coefficient and "n" is a constant that stands for the power factor of the transition mode. However, the value of "n" depends on the photon transmission and properties of the material being used. Therefore, some values are used for certain transitions "n," i.e., direct allowed (1/2), directly forbidden (3/2), indirectly allowed (2), and indirectly forbidden (3)^48,49^. In this work, the value of direct transition is 3.9, 3.7, 3.4, and 3.3 eV for 4:1, 1:1, 2:1, and 1:2, respectively Fig. 1b.

**Figure 2:**
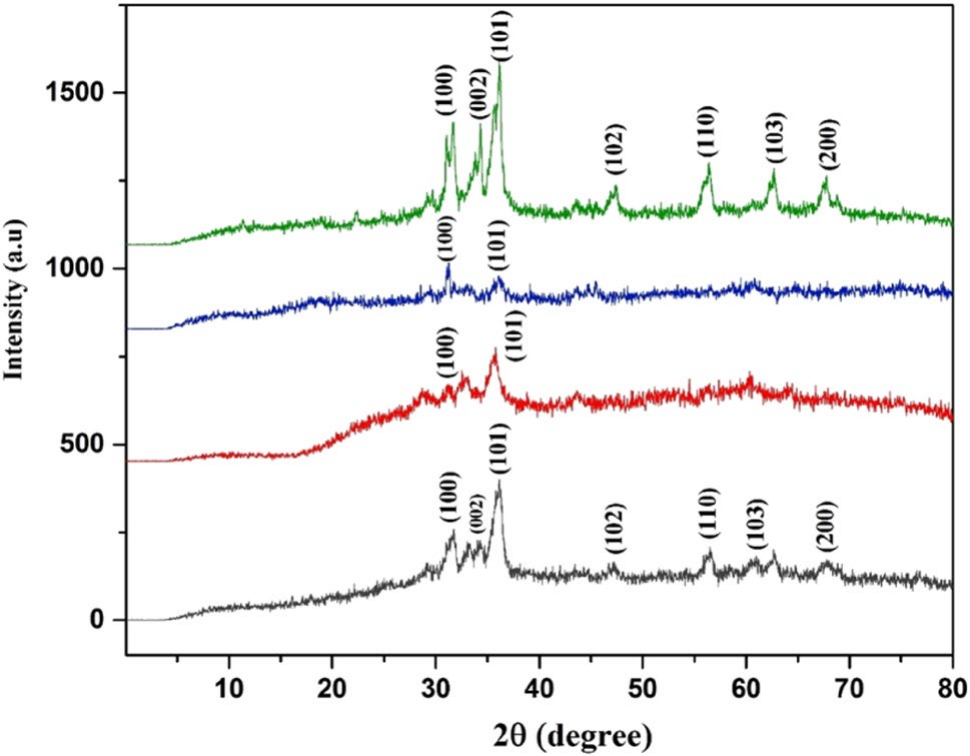
XRD analysis of manganese doped zinc with different precursor ratios 4:1, 1:1, 2:1, and 1:2 (Green, Blue, Red, Black).

XRD analysis was used in an experiment to determine the nanoparticle's structural properties and their synthesis. The XRD pattern of green synthesized Mn-doped ZnO nanocomposites was measured within 20 to 80^o^ C. The strong and narrow diffraction peaks show that Mn-doped ZnO nanocomposites were synthesized and characterized previously^50^. From Fig. 2, it was shown that diffractograms of the green synthesized Mn-doped ZnO nanocomposites having different ratios of compositions Mn-doped ZnO nanoparticles showed the sharp and clear peaks at 31.7, 34.3, 36.1, 47.2, 56.4, 60.7, 63.2 shown at the (100), (002), (101), (102), (110), (103), (200), respectively. The hexagonal structure of Mn-doped ZnO angles and its planes correspond to the JCPDS Card no. 00-001-1136. These outcomes confirm that the amorphous tetragonal structure of Mn-doped ZnO nanocomposites by green synthesized with help of *W. coagulans* acts as a reducing agent and shows no presence of another impurity. Our research results can be compared with the previous report in which fabrication of different nanomaterials and their different applications^51^. The biologically fabricated nanoparticle peaks are wider at the lower position, indicating that the crystalline size is small. The calculated crystalline size was 14, 11, 12, 12 nm for 2:1, 1:1, 1:2, and 4:1, respectively black, blue, red, green in Fig. 2.

**Figure 3:**
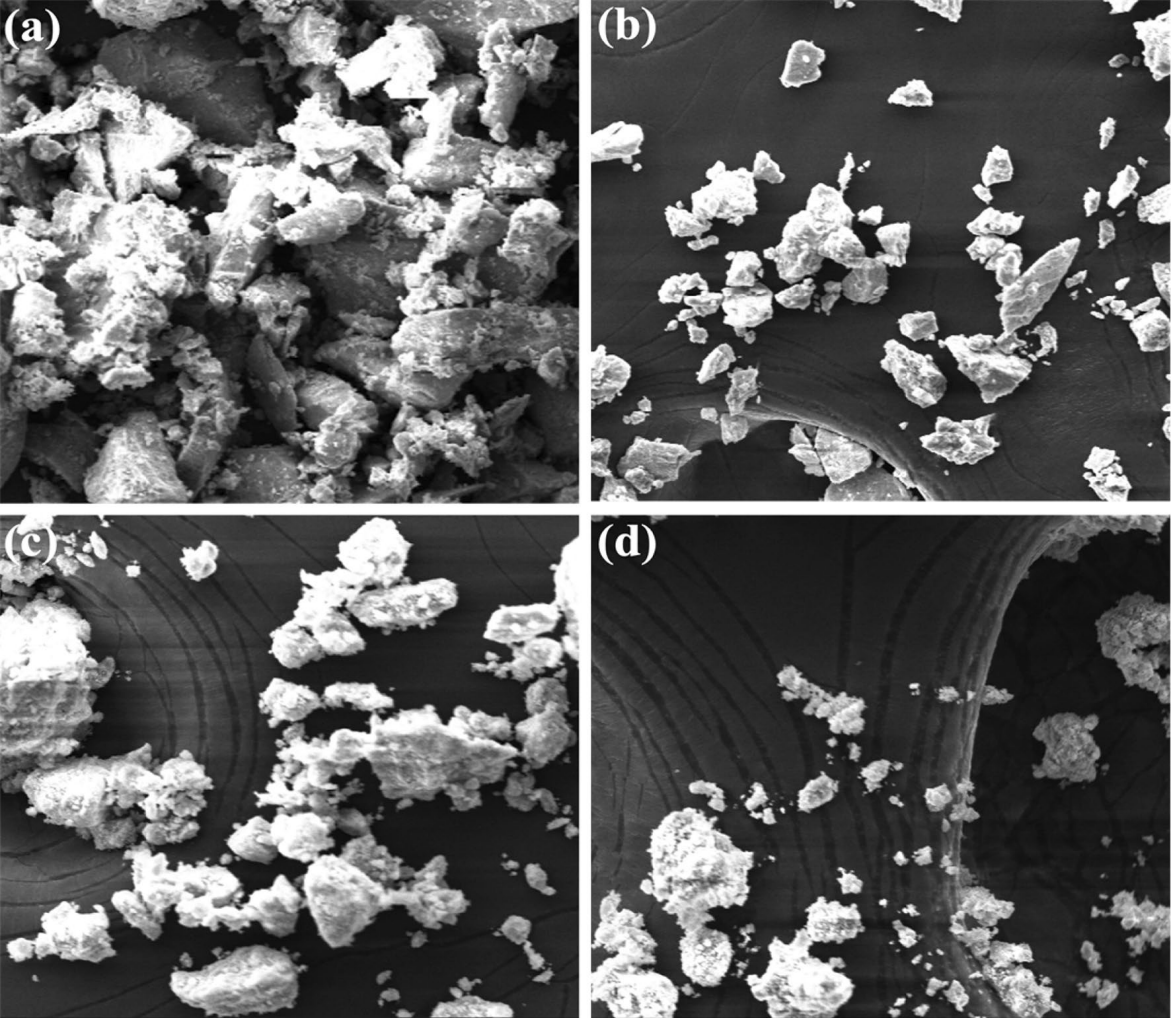
SEM images of Mn-doped ZnO nanoparticles of different concentrations (**a**) 4:1, (**b**) 2:1, (**c**) 1:1 (**d**) 1:2 Mn-doped ZnO Nanocomposite respectively.

Scanning electron microscopy is one of the tools used for detail information regarding the morphology and surface chemistry and attachment of bioreducing agent on it. Here in this study, the observation was done to investigate the surface structure of Mn-doped ZnO nanocomposites^51,52^. The results show the amorphous tetragonal structural morphology of the nanocomposites as in Fig. 3a–d. There is clearly difference in the morphology and shapes of samples due to various manganese and ratio mixed with the zinc oxide nanoparticles. Figure 3a illustration is best due to maximum concentration of manganese 4:1. While the other ratio was also works properly but shows less optimized morphology. The main difference is manganese concentration while the plant extract presence is also a key role for reducing and giving it final shapes of nanocomposites^53,54^. The surface characteristics of the synthesized Mn-doped ZnO nanocomposite are obeyed by a series of secondary metabolites that induce more catalytic sites. The phytochemical-assisted morphology of the Mn-doped ZnO nanocomposites infers a less agglomerated and coarse surface. The Mn-doped ZnO showed well-formed tetragonal symmetry that has been observed with varying optimization processes of capping with plant extract. Additionally, it can be observed that each Mn-doped ZnO particle characteristic becomes distinguishable after encapsulation of plant phytochemicals. The smaller size makes it easier for the nanocomposite to penetrate through the toxic organic materials and microbes, leading to the cell decomposition process.

**Figure 4:**
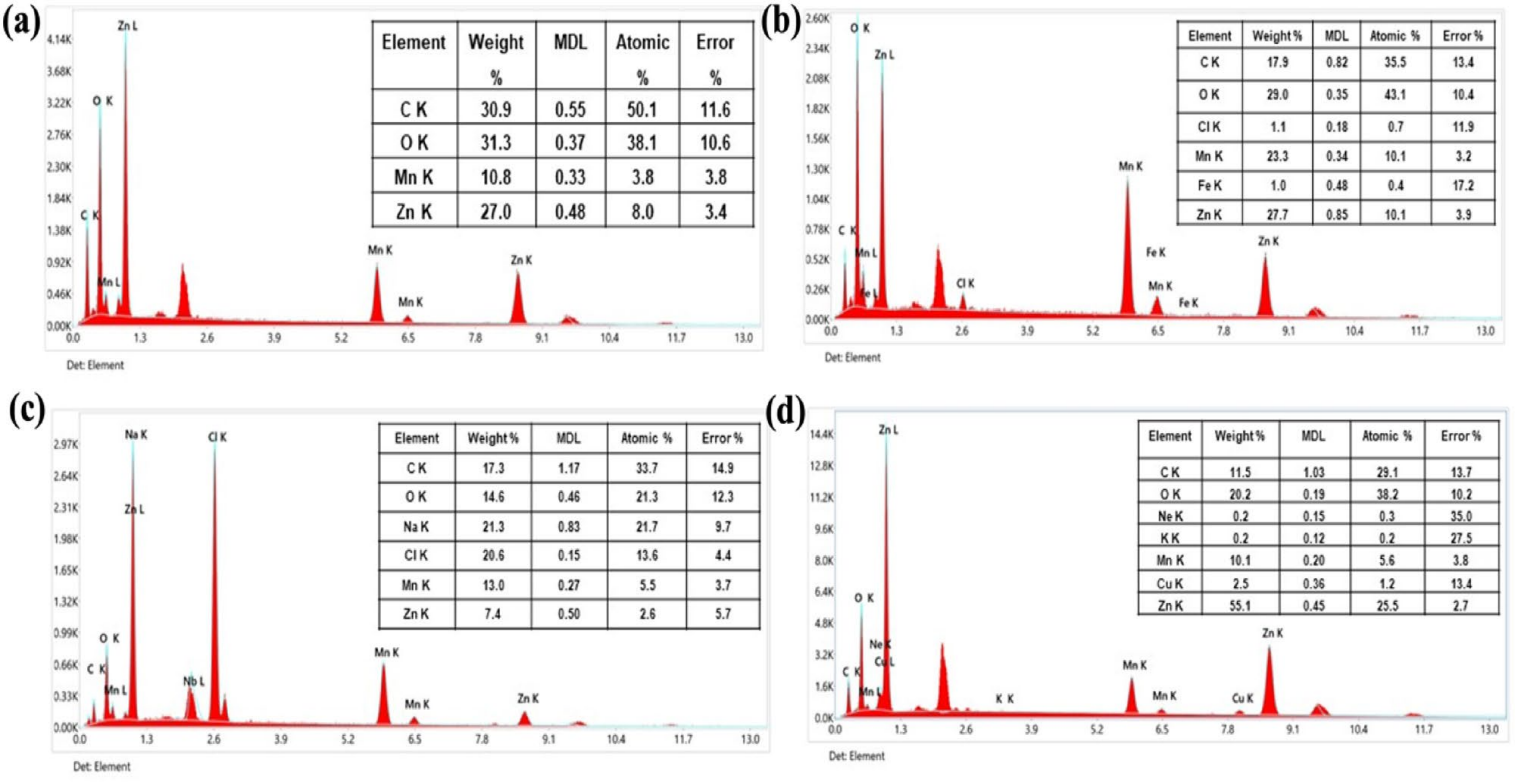
EDX images of Manganese-doped Zinc of different concentrations (**a**) 4:1, (**b**) 2:1, (**c**) 1:1 (**d**) 1:2 Mn-doped ZnO Nanocomposite respectively. Ftir specta Mn-doped ZnO Nanocomposite sjows various bonds.

Energy dispersive x-ray spectroscopy analysis was carried out to determine the elemental analysis of the intricated composition of *W. coagulant*-based Mn-doped ZnO nanocomposite. All the samples biologically synthesized with various concentrations of Mn doped ZnO formulating nanocomposite have the peaks of Zn, Mn, C, and O, sodium, calcium, carbon, hydrogen. The presence of trace elements in different forms indicating the presence of biological materials comes from bioreducing agent which confirmed our green synthesis process Fig. 4a–d. The sharp peaks of zinc and manganese in all samples also confirmed that Mn-doped ZnO nanocomposites were produced as reported in the previous work^55–57^.

### Antibacterial activity

**Figure 5:**
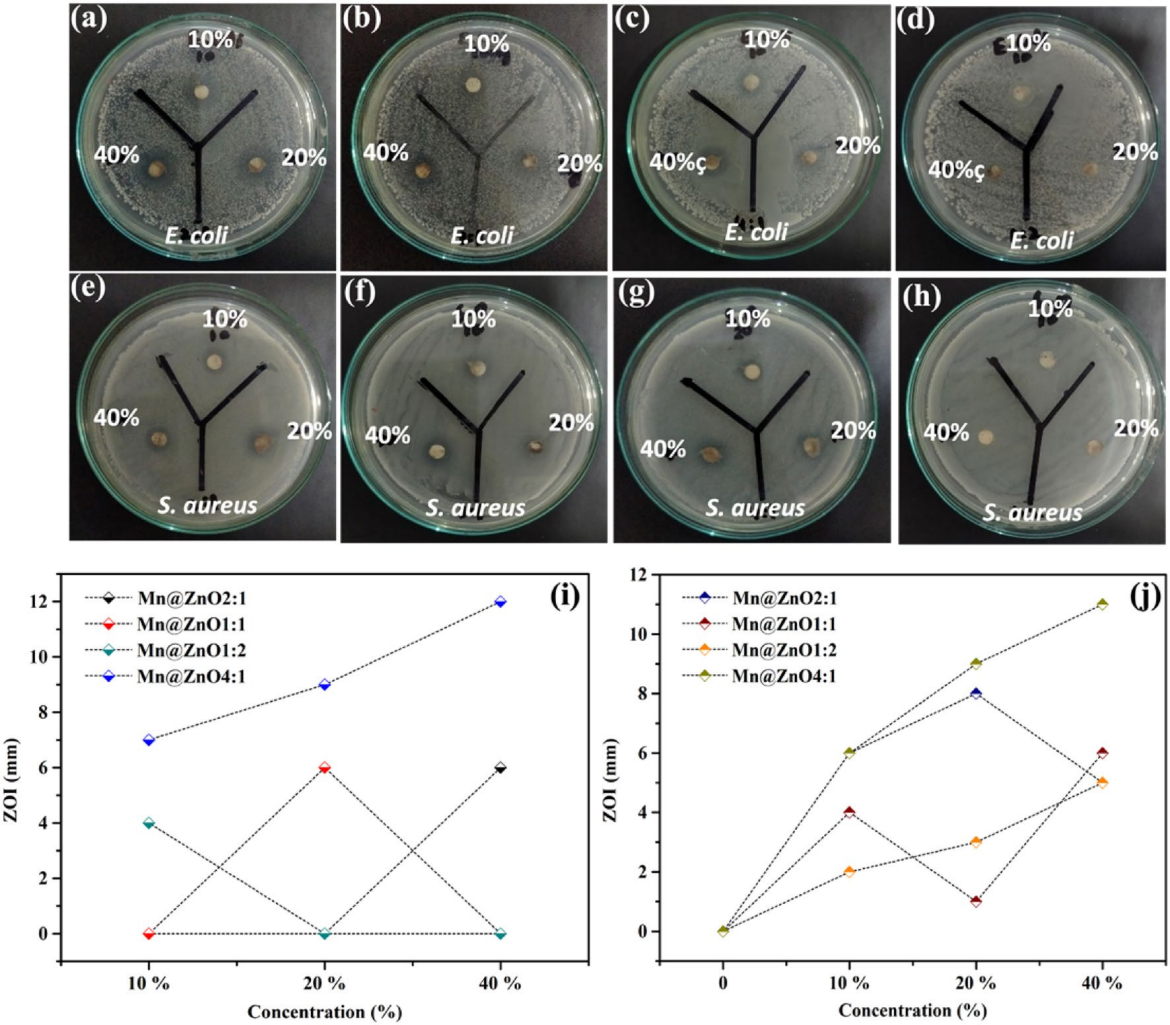
Antibacterial Activity of Different ratio of Mn-doped ZnO nanocomposites against (**a**–**d**) *E. coli,* (**e**–**h**) *S. aureus* (**i**) Graphic presentation of Inhibition zone against *E. coli* (j) *S. aureus*.

In this work, using the disc diffusion method*, W. coagulans-*based nanoparticles Mn doped ZnO show antibacterial activity against the *E. coli* (rods) and *S. aureus* (cocci). The zone of inhibition differs from species to species of bacteria. In this experiment, different dilutions of biologically synthesized Mn-doped ZnO nanocomposites with different percentage were used as compared to control. It was observed in the current studies that changing the percentage and ratios of manganese with zinc doped process has changed the antibacterial process. Different concentrations of 10%, 20 and 40 μg/mL of biologically prepared nanocomposites were used. It was observed that the growth of *S. aureus* showed more fluctuation on all concentration as compared *to E coli.* Every sample showed deviations from each zone of inhibitions. The results showed it as the concentration of Mn-doped ZnO composite increases, the zone of inhibition also increases Fig. 5a–h. Moreover, 40% concentration with nanocomposites showed more zone of inhibition in *E coli* Fig. 5i whereas 20% concentration with nanocomposites showed lower zone of inhibition in *S. aureus* Fig. 5j.

The given results showed maximum zone of bacterial inhibition (ZOI) is that Mn-doped interacts with the negative surface of the bacterial membrane, which caused a change in potential gradient. The disturbance in the cellular environment towards the surface charged due to manganese and zinc-positive ions that altered the permeability or disturbed the structure of the membrane^58–60^. The excessive production of highly reactive oxygen species, including H_2_O_2_, OH radicals, and singlet oxygen, causes a disturbance in the cellular membrane and leads to bacterial death^61–63^. It is inferred that green synthesized Mn-doped ZnO nanocomposites are good antibacterial catalysts against resistant bacterial strains.

### Photocatalytic activity

**Figure 6:**
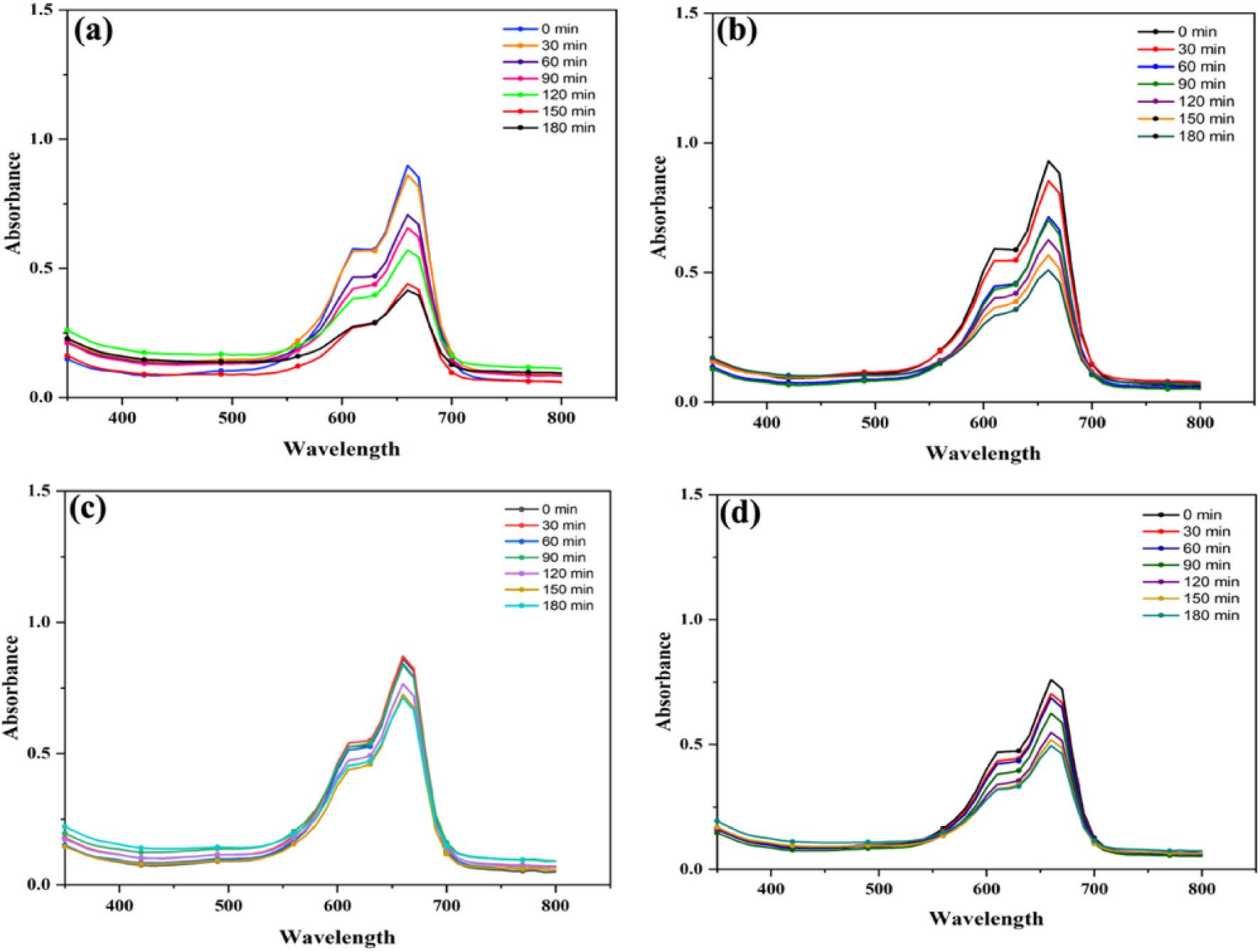
Photocatalytic degradation of Mn-doped ZnO Nanocomposite with Different concentration (**a**) 4:1 (**b**), 2:1 (**c**), 1:1 (**d**), 1:2.

The effectiveness of photocatalytic degradation was evaluated for an amorphous tetragonal nanocomposite of Mn-doped ZnO, which was fabricated using green methods. To test its efficiency, the pollutant methylene blue dye was used as a sample, and the process was conducted under neutral pH conditions Fig. 6a–d. After degradation activity solution was collected and its concentration before and after degradation by using all four different ratios of manganese doped zinc was measured using UV–Vis spectrophotometer at various time intervals^64,65^. When the MB dye is degraded in the presence of sunlight and sample gives two sharp peaks with high intensity at 660 nm and 610 nm. As the degradation proceeds, it is verified that the bond between nitrogen-sulphur is broken in the dye and the peak at 610 nm goes steeper and later vanishes as time moves further^36,60, 66^. The solution was taken and preserved for UV analysis in the dark at different intervals with a gap of 30 min. It is observed and studied in the previous work that the photocatalysis procedure under the presence of sunlight and catalysts produces CO_2_ and H_2_O as a result of byproducts after the oxidation of organic matter^67,68^. The Mn-doped ZnO nanoparticles with a ratio of 4:1 have a degradation of 53.8% over time The degradation efficiency is calculated by the formula:

$$\mathrm{Removal\, Efficiency\%}=\left[\left(\frac{{A}_{o-{A}_{i}}}{{A}_{i}}\right)\right]\times 100\%.$$

As the degradation proceeds, the color of the MB dye solution which was very darker becoming less dark with the passage of time under the presence of light and photocatalyst. The degradation of the methyl blue was observed due to the electrons (e^−^) and holes (h^+^) generated, and they react with metals and form heterojunction formation to degrade these dyes by producing hydrogen peroxide and hydroxyl radicals^69,70^. It was discovered that adding extra surface defects to particles' surfaces increases their porosity, which enhances their ability to absorb light and strengthen their photocatalytic degradation activity^47,67, 71^. The Mn-doped ZnO nanocomposite with the ratio of 4:1, 1:1, and 1:2 shows degradation activity of 53.6%; 45.16%, 18.2%, and 34.7%, respectively against the methylene blue dye (Fig. 6b–d). During the degradation, it gives two peaks at 610 nm and 660 nm Fig. 6a–d. Our work is related to the previous studies that the charge of ZnO is increased by adding the manganese oxide metal, which establishes the heterojunction formation between them and makes the nanocomposite more powerful, cheap, requires less time for degradation, stable, reusable and eco-friendly to degrade the dye^72–74^.

This table explains that the 2:1 nanocomposite showed the highest degradation of dye, 53.8%, while the least is 18.2% by 1:1 Mn-doped ZnO nanoparticles.

**Figure 7:**
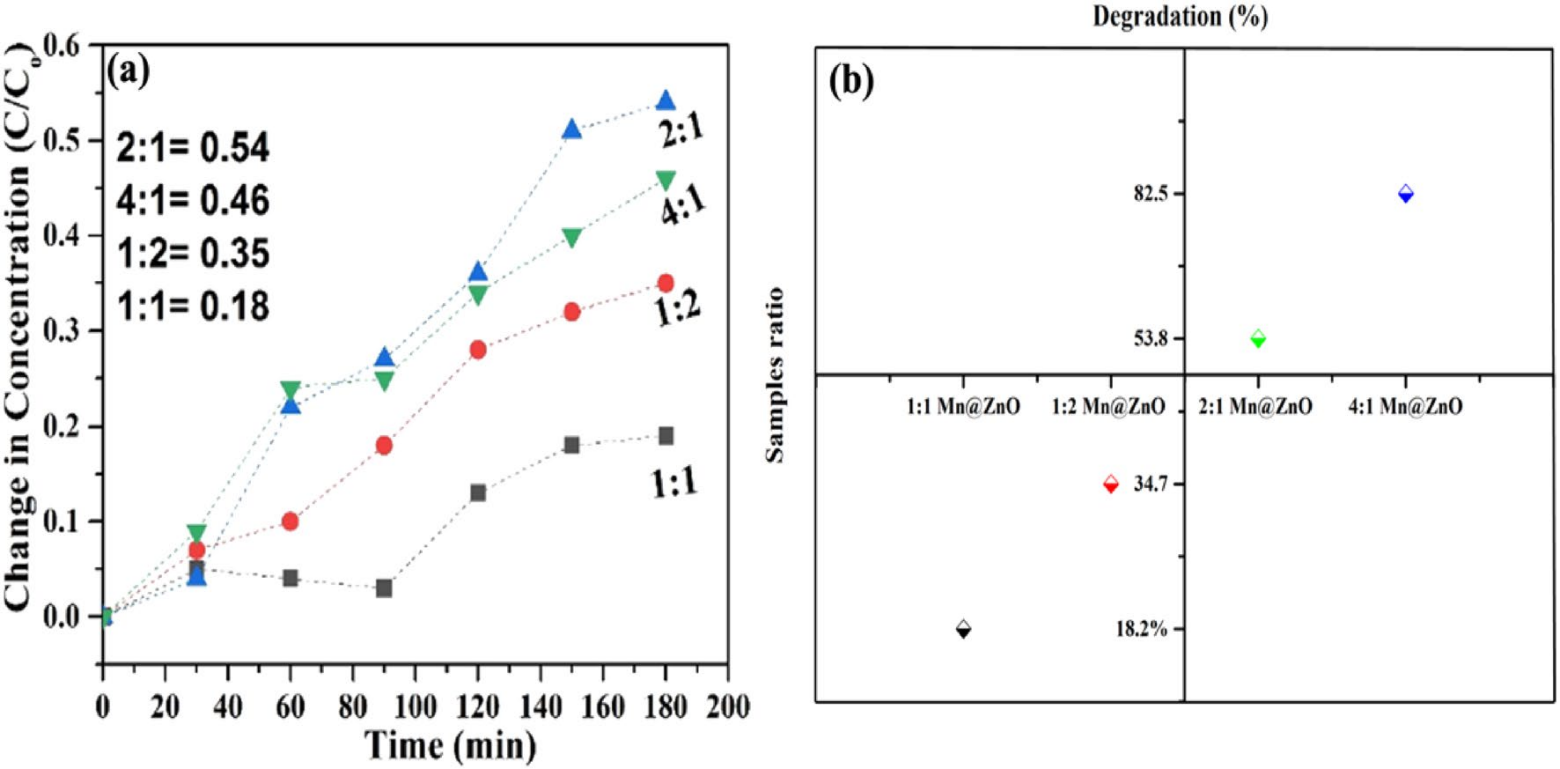
Photocatalytic Degradation of Mn-doped ZnO Nanocomposite (**a**) C/C_o_ vs. irradiation time for Methyl Blue dye degradation (**b**) Percentage degradation.

Figure 7a demonstrates the difference between the ratios and the dye degradation at different time intervals. The dye is gradually degraded from 0 to 0.54 in 180 min under the presence of 2:1 Mn doped ZnO nanoparticles, 0 to 0.46 in 180 min under the presence of 4:1 Mn doped ZnO nanoparticles, 0 to 0.35 in 180 min under the presence of 1:2 Mn-doped ZnO nanoparticles and 0 to 0.18 in 180 min under the presence of Mn-doped ZnO nanoparticles. The 2:1 Mn-doped ZnO nanoparticles show higher degradation of the dye. similarly, Fig. 7b the degradation of dye by different manganese with zinc also showed in percentage form.

## Conclusions

Mn-doped ZnO nanoparticles are prepared by using the reducing and capping components of the plant extract *W. coagulans*. The basic purpose of this research is to fabricate, characterize and biologically evaluate the Mn-doped ZnO nanoparticles. By doing the XRD, it confirms the nanoparticle's crystalline stability and its smaller size 11–14 nm. The EDX of different ratios 2:1, 4:1, 1:1, 1:2 of Mn doped ZnO nanoparticles gives the outcomes of 27.0%, 10.8%, 55.1%, 10.1%, 27.7%, 23.3%, and 7.4%, 13.0% respectively. The antibacterial activity was performed, showing more activity against the gram-positive bacterial strain *S. aureus* and less against the gram-negative bacteria *E. coli*. Overall, it was shown that biologically fabricated nanoparticles are more stable, crystalline, smaller, and active. Additionally, they showed greater antibacterial and photocatalytic activity against the bacteria and dye, respectively. Mn-doped ZnO nanoparticles efficiently derived from the biological source with aim to minimize the expenditure of hazardous chemicals. Controlled synthesis of Mn-doped ZnO, eco-friendly, biocompatible, stable, and their potential desirable properties make it a strong nanotechnological advancement candidate. So, Mn-doped ZnO nanoparticles can be used further in various biomedical and water treatment industries for a clean and pure environment.

### Supplementary Information

Supplementary Information.

## Data Availability

All the data generated/ analyzed during the study are available with the corresponding author on reasonable request.
