# Supplementary material for: A comparative study on green synthesis and characterization of Mn doped ZnO nanocomposite for antibacterial and photocatalytic applications
Source: Sci Rep. 2024 Mar 29;14:7528. doi: 10.1038/s41598-024-58393-0 (PMC10980724; doi:10.1038/s41598-024-58393-0)
Supplement: Supplementary file 1 — Supplementary Information. [file 41598_2024_58393_MOESM1_ESM.docx]

**Experimental Section**

After this 20 g crushed powder was measured and soaked in the 200 mL distilled water and boiled for 2 hours on a hot plate at 90^o^C. After heating, the mixture was cooled at ambient temperature to get the extract and this extract after filtration was stored at 4^o^C in a refrigerator for further studies.


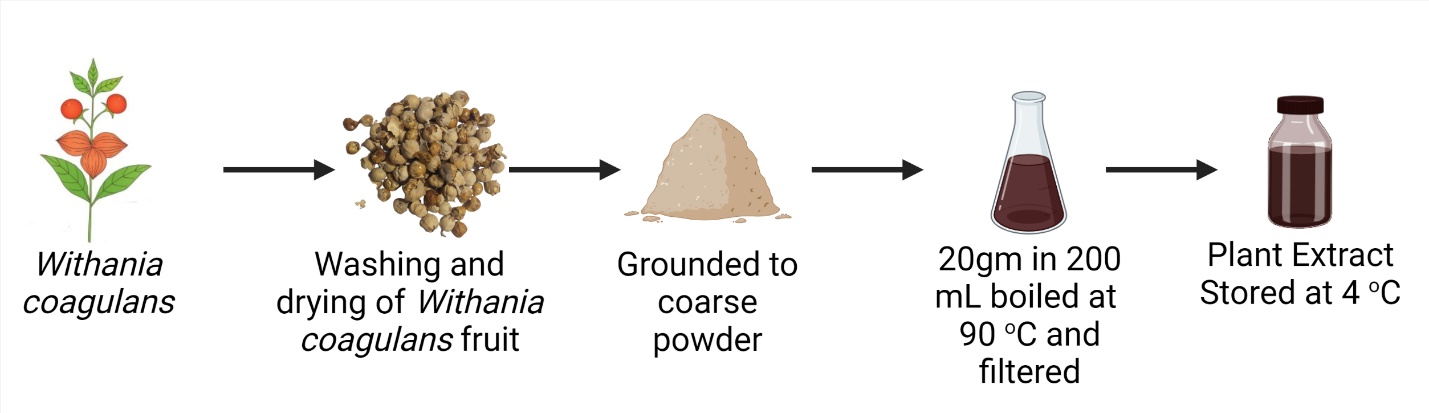


Fig. S1. Preparation of *Withania coagulans* Extract as reducing agent

.


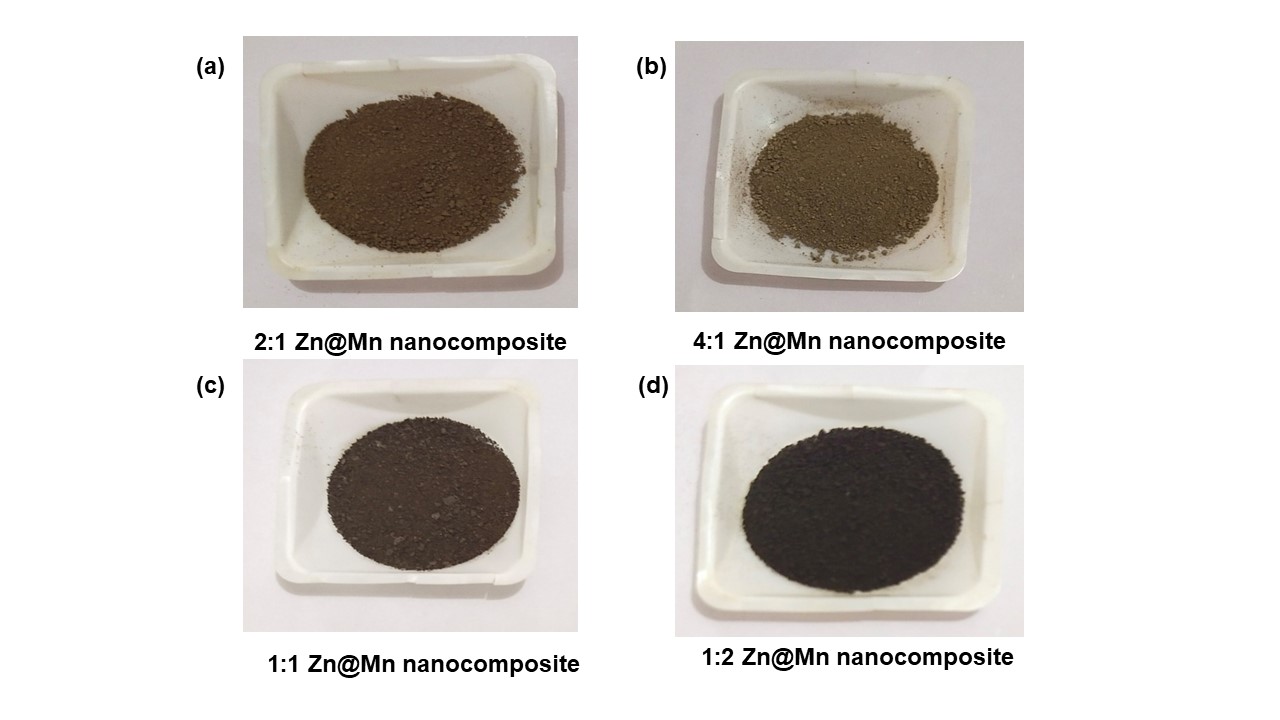


Fig S2. Resulting synthesized of Mn-doped ZnO nanocomposites ratios


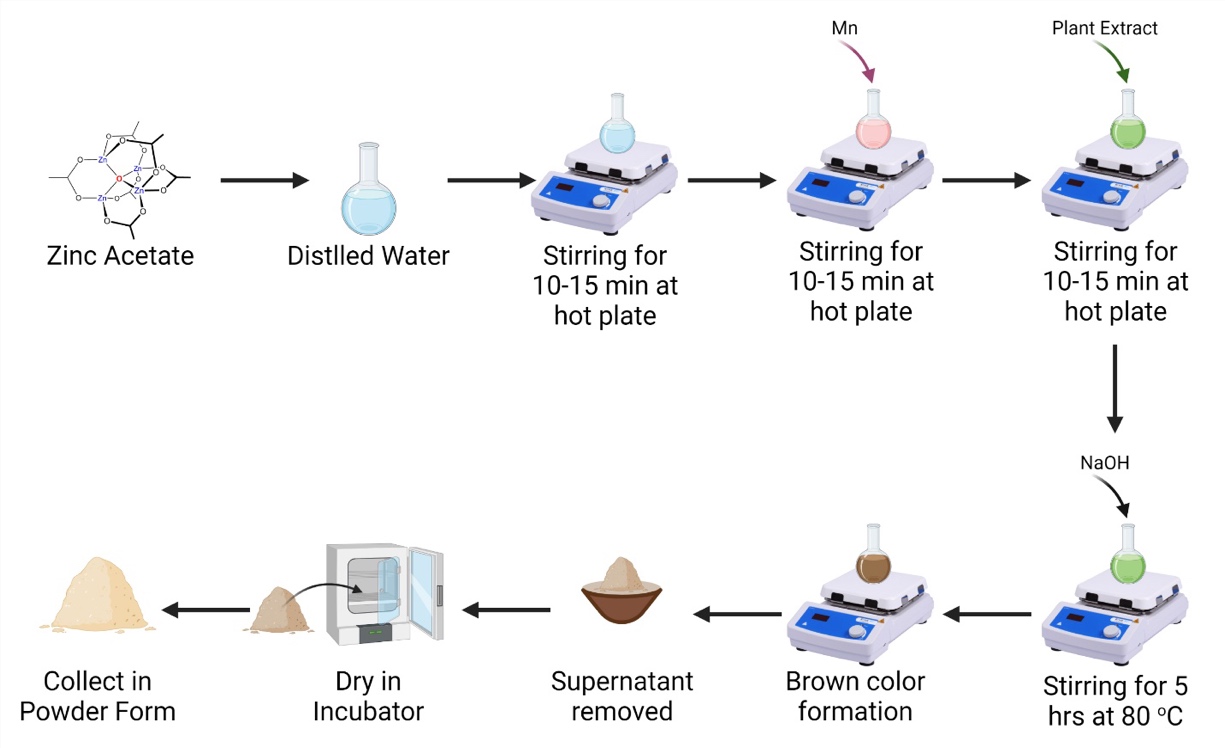


Fig S3. Preparation of Mn-doped ZnO nanocomposites
